# Incidence rates of tuberculosis in chronic hepatitis C infected patients with or without interferon based therapy: a population-based cohort study in Taiwan

**DOI:** 10.1186/s12879-014-0705-y

**Published:** 2014-12-19

**Authors:** Shang-Yi Lin, Tun-Chieh Chen, Po-Liang Lu, Chun-Yu Lin, Wei-Ru Lin, Yi-Hsin Yang, Yen-Hsu Chen

**Affiliations:** Kaohsiung Municipal Ta-Tung Hospital, Kaohsiung Medical University Hospital, Kaohsiung Medical University, Kaohsiung, Taiwan; School of Medicine, Graduate Institute of Medicine, Sepsis Research Center, College of Medicine, Kaohsiung Medical University, Kaohsiung, Taiwan; Division of Infectious Diseases, Department of Internal Medicine, Kaohsiung Medical University Hospital, 100 Tzyou 1st Road, Kaohsiung City, Taiwan; Department of Biological Science and Technology, College of Biological Science and Technology, National Chiao Tung University, Hsinchu, Taiwan; Department of Laboratory Medicine, Kaohsiung Medical University Hospital, Kaohsiung, Taiwan; School of Pharmacy, Kaohsiung Medical University, Kaohsiung, Taiwan; Division of Statistical Analysis, Department of Medical Research, Kaohsiung Medical University Hospital, Kaohsiung, Taiwan

**Keywords:** Interferon, Hepatitis C virus, Tuberculosis

## Abstract

**Background:**

It is debated whether interferon-based therapy (IBT) would affect the incidence of active tuberculosis (TB) among hepatitis C virus (HCV) infected patients. Although some case reports have demonstrated a possible association, the results are currently inconclusive. Therefore, we conducted a nation-wide population study to investigate the incidence of active TB in HCV infected patients receiving IBT in Taiwan.

**Methods:**

This 9-year cohort study was based on the Longitudinal Health Insurance Database 2000 (LHID 2000) consisting of 1,000,000 beneficiaries randomly selected from all Taiwan National Health Insurance enrollees in 2000 ( >23.7 million). This insurance program covers all citizens in Taiwan. We conducted a retrospective cohort study that identified subjects with HCV infection. IBTs were defined as regimens that included interferon α, peginterferon α2a and peginterferon α2b for at least 2 months. Among them, 621 subjects received IBT, and 2,460 age- and gender-matched subjects were enrolled for analysis. The Cox proportional hazards models were used to estimate the hazard ratio (HR) for active TB, and associated confidence intervals (CIs), comparing IBT cohort and untreated cohort. The endpoint in this study was whether an enrolled subject had a new diagnosis of active TB.

**Results:**

During the 9-year enrollment period, the treated and untreated cohorts were followed for a mean (± SD) duration of 6.97 ± 0.02 years and 8.21 ± 0.01 years, respectively. The cumulative incidence rate of active TB during this study period was 0.150 and 0.151 per 100 person-years in the IBT treated and untreated cohorts, respectively. There was no significant difference in the incidence of active TB in either cohort during a 1-year follow-up (Adjusted Hazard Ratio (AHR): 2.81, 95% Confidence Interval (95% CI): 0.61–12.98) or the long-term follow-up (AHR: 1.02, 95% CI: 0.28 – 3.78). The Cox proportional hazards model demonstrated that IBT was not a risk factor for active TB . The only risk factor for active TB was the occurrence of hepatic encephalopathy.

**Conclusion:**

Our results showed that IBT is associated with increased hazard of active TB in HCV infected patients in 1-year follow-up; however, the effect sizes were not statistically significant.

**Electronic supplementary material:**

The online version of this article (doi:10.1186/s12879-014-0705-y) contains supplementary material, which is available to authorized users.

## Background

An estimated 170 million people are chronically infected with Hepatitis C virus (HCV), and 3 to 4 million people are newly infected each year [1]. HCV infection is the major cause of chronic liver disease worldwide and can lead to cirrhosis and hepatocellular carcinoma [1]. Therefore, effective anti-HCV therapies are recommended by national and international guidelines [2]. Pegylated interferons plus ribavirin have been used as the standard treatment of HCV infection for decades [3],[4].

Interferons (IFNs) are a group of structurally related cytokines that are important in antiviral activities [5]. There are two types of IFNs, type I and type II. Type I IFNs, such as IFN-α/β, are produced in most cell types in response to microorganism infections and play a crucial role in innate immune activity against viruses [6]. The type II IFN, INF-γ, is the main mediator of the type I immune response and is essential in the control of mycobacterial infection in both animal models and humans [7]. In contrast to IFN-γ, the role of INF-α/ß in tuberculosis (TB) is controversial. In an *in vitro* study, IFN-ß was shown to improve bacillus Calmette-Guerin (BCG) immunogenicity by increasing human dendritic cell maturation [8]. In an animal model, type I IFN limited the number of target cells that *M.tuberculosis* infected in the lungs [9]. Early clinical pilot studies demonstrated that aerosolized IFN-α combined with standard therapy for pulmonary TB would lead to better clinical outcomes [10],[11]. However, some studies showed that type I IFNs promoted, rather than inhibited, mycobacterial infection. In an *in vitro* model, type I IFNs impaired the ability of human macrophages to control the growth of *M. bovis* BCG and *M. avium intracellulare* complex [12],[13]. In an animal model, Manca *et al*. reported that type I IFNs enhanced the virulence of *M. tuberculosis* by the suppression of the Th1 type immune responses. In addition, the treatment of TB infected mice with IFN-α/β increases lung bacterial loads, resulting in reduced survival [14]. Together, these studies indicate that the role of type I IFNs in mycobacterial infections is still inconclusive.

The side effects of IFNs include fatigue, influenza-like symptoms, hematological abnormalities, and neuropsychiatric symptoms [15]. Pulmonary manifestations, including sarcoidosis, interstitial pneumonitis and bronchiolitis obliterans organizing pneumonia, are considered rare events [16],[17]. Although altered cellular immunodeficiency is associated with a higher incidence of various infections, TB has rarely been reported during HCV treatment [18]-[22]. This may be because the people receiving interferon-based therapy (IBT) for HCV were mostly located in countries with low TB incidence rates; this creates a difficulty in identifying an association between IBT and active TB.

Taiwan is a hyperendemic area of chronic liver diseases and has an HCV seroprevalence ranging from 0.4 to 10.5%, depending different geographic areas [23]. Because HCV infection can lead to fetal comorbidity, the Bureau of Taiwan National Health Insurance (NHI) has reimbursed IBT since 2003. Taiwan is also an endemic TB area with an intermediate burden of TB. In 2008, 2009 and 2010, the incidence rates of TB in Taiwan were 57.8, 57.2 and 54.5 per 100,000 population, respectively [24]. Therefore, this study used a longitudinal Health Insurance Database 2000 (LHID 2000) that included a nationally representative population, and used an epidemiological approach to evaluate whether IBT is a risk factor for the development of active TB during January 2000 to December 2009.

## Methods

### Study sample

National Health Insurance (NHI) is a single-payer compulsory program that has been implemented in Taiwan since 1995 and covers all forms of health care for the residents of Taiwan [25]. All citizens who have established a registered domicile for at least 4 months in the Taiwan area should be enrolled in the NHI. There are approximately 23,720,000 individuals in this program. The NHI comprehensively includes a claims database, including ambulatory care, outpatient services, inpatient services and prescription drugs.

We used a database (LHID2000) containing one million randomly selected subjects from the Taiwan National Health Insurance Research Database (NHIRD), which was developed for research purposes. A systematic, random sampling method was used to build this representative database of 1,000,000 NHI enrollees. There were no statistically significant differences in age, sex, or healthcare costs between the sample group and all the enrollees. This data set spans from January 2000 through December 2009 and includes all claims data for these 1,000,000 individuals.

### Ethics statement

The identification numbers of all of the subjects in the NHRI databases were encrypted to protect the privacy of the individuals. All researchers who used the NHIRD and its data subsets were required to sign a written agreement declaring that they had no intention of attempting to obtain information that could potentially violate the privacy of patients or care providers. This study was approved by the Institutional Ethics Review Board of Kaohsiung Medical University Hospital (Kaohsiung, Taiwan) (IRB No 20130067).

### Study design and population-based surveillance methods

This study used a retrospective cohort study design to evaluate the association between IBT and TB events. This study enrolled patients who were 20 or older from this database (LHID2000).

The Taiwan NHIRD did not contain direct laboratory results (such as biochemical data, viral genotype, viral load, histological characteristics). Therefore, we were unable to identify the HCV infected patients based on laboratory diagnostic criteria. With approval from the NHRI, we were able to use the scrambled patient identification numbers to interlink files, including registry of medical facilities, details of inpatient orders, ambulatory care, and prescriptions.

The definition of HCV infection was based on individuals who had at least two service claims of ambulatory or inpatient care for the treatment of HCV between 2000 and 2008. For only once service claim (The diagnosis coding of NHI in Taiwan is performed according to the International Classification of Disease, 9th Revision, Clinical Modification (ICD-9-CM) diagnostic criteria.) would overestimate the diagnosis of HCV. Therefore, we used at least two service claims of ambulatory or inpatient treatment care to identify this group. (HCV infected patients) [26]. We defined HCV by compatible ICD-9-CM codes of HCV in 070.41, 070.44, 070.51, 070.54, and V02.62.

**Figure 1:**
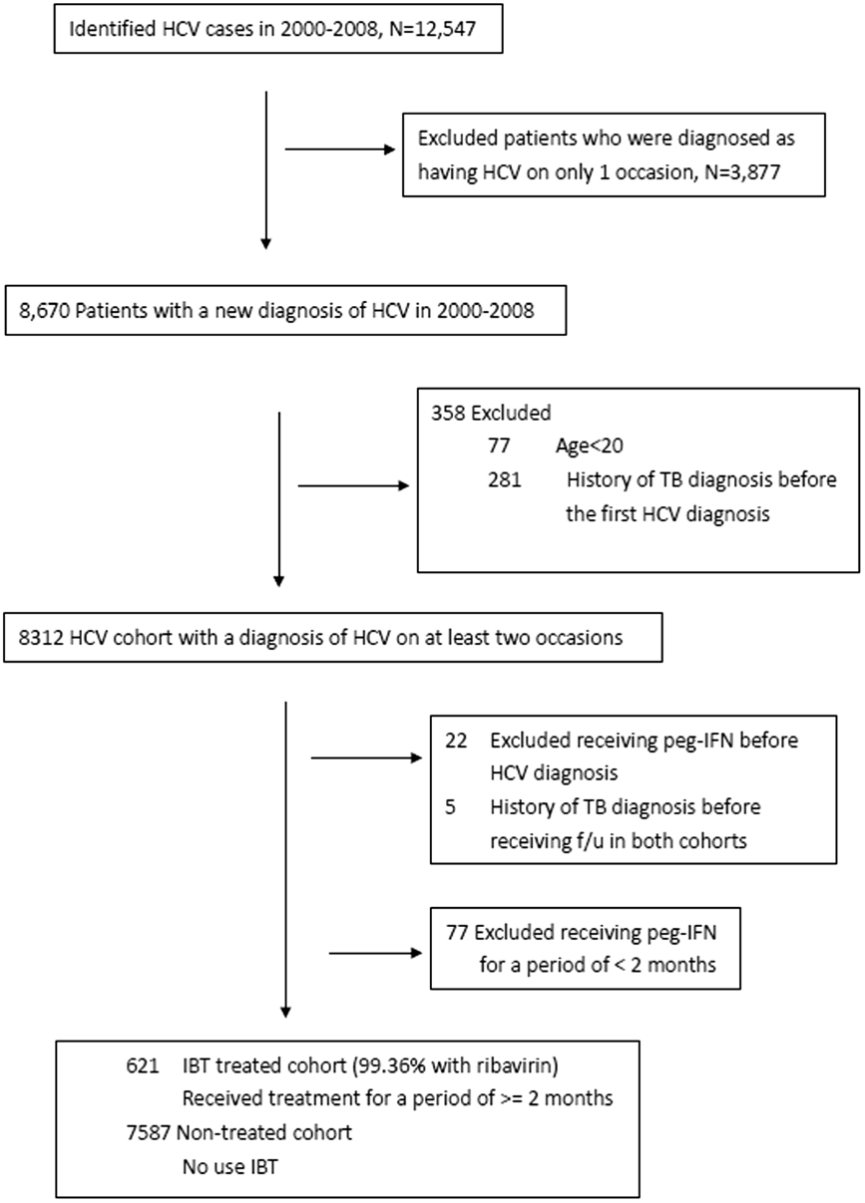
**Study flow chart for the enrollment of participants.**

A total of 12,547 subjects with ICD-9-CM codes of HCV were identified. Furthermore, we excluded patients who were diagnosed as having HCV on only 1 occasion (n = 3,877), who were below 20 years old (n = 77) and who had a history of TB diagnosis codes (ICD-9 code:010–018) before the first HCV coding and at the same time as the first HCV coding (n = 281). Therefore, 8,312 patients with a diagnosis coding of HCV on at least two medical claims were enrolled (Figure 1).

### Definition of study cohorts: cohorts treated and not treated with IBT

Combination regimens with peginterferon α (either 2a or 2b) and ribavirin have been introduced for treating HCV infection in Taiwan since October 1, 2003. Generally, antiviral therapy was initiated as peginterferon α2a (180 μg per week, irrespective of body weight) or peginterferon α2b (1.5 μg /kg per week) combined with ribavirin (800 to 1,200 mg per day). The duration of treatment ranged from 16 to 48 weeks, depending on viral genotype, serum viral load, clinical response, and patient tolerability [4].

The treated cohort was comprised of individuals who had received IBT; these individuals were identified from the database using the drug codes for interferon α, peginterferon α2a and peginterferon α2b. Most of the subjects (99.36 %) had received interferon combined with ribavirin treatment in this study. Patients who had received IBT before HCV diagnosis coding index date were excluded (n = 22). Because NHI in Taiwan reimbursed chronic HCV infected patients 4–6 months of interferon or 6 months of pegylated interferon-based treatment during 2000–2008, and most patients who did not achieve early virologic response after 3 months of treatment discontinued IBT [3],[4],[27], we selected patients receiving ≧2 months of IBT into our analyses(IBT treated cohort) and patients who had received IBT for a period of <2 months were excluded (n = 77). For those patients who had received IBT≧ 2 months (n = 621) between 2000 and 2008 were grouped into the treated cohort. The first day of prescription use for the treatment of HCV was assigned as the index date.

Those patients who did not receive IBT between 2000 and 2008 were designated as the non-treated cohort (n = 7587). We selected 2,460 control subjects from the non-treated cohort. 95.98% of the IBT treated subjects were matched at a ratio of 1:4 with the non-treated cohort in terms of age, sex and the year and month of the index visit. The control subjects did not include patients who had TB before their index date. Furthermore, patients who had a diagnosis of TB before receiving follow-up in both cohorts were excluded (n = 5).

### Study endpoints and adjustment for confounding factors

The endpoint in this study was whether an enrolled subject had a new diagnosis of TB. The IBT and non-treated cohorts were both tracked from the date of selection until the end of 2009 or until loss to follow-up (i.e., withdrawing from the health insurance program) to identify new TB events.

### The definition of active TB

We defined active TB as at least one outpatient visit or one hospital admission during the follow-up period with ICD-9-CM codes of TB (010–018) plus the prescription of more than two anti-tuberculosis medications (i.e., isoniazid, rifampin, pyrazinamide, ethambutol, rifater, rifinah, streptomycin, cycloserine, prothionamide, amikacin, kanamycin, ciprofloxacin, moxifloxacin, and levofloxacin) for more than 90 days during the study period [28],[29].

It is possible that patients with other diseases (e.g., nontuberculous mycobacterial infection, lung cancer, or latent TB infection) were misdiagnosed with active TB and put on anti-tuberculosis medications initially. To avoid this misclassification of outcome, we screened the NHI records of patients who were classified as active TB cases by this definition. If the ICD-9 codes of TB (010–018) in these patients were replaced by those of nontuberculous mycobacterial infection (031), lung cancer (162), or positive tuberculin skin test (795.5) during subsequent follow-up with discontinuation of anti-tuberculosis medications, the patients would be reclassified as non-TB.

### Confounders

To determine the impact of IBT on the risk of active TB, it is important to take into consideration the influences of known risk factors, such as HIV infection (ICD-9 code 042), silicosis (ICD-9 codes 501–504), diabetes (ICD-9 code 250), chronic obstructive pulmonary disease (COPD) (ICD-9 codes 403, 416, 491–493, 495–495,508, 515, 516, and 518), connective tissue disease (ICD-9 codes 710, 712, 714, 715, 716, 719, and 728), End Stage Renal Disease (ESRD) (ICD-9 code 585.6) and malignancy (ICD-9 codes 140–239) [30]. Because smoking status was unavailable for this database, COPD was selected as a proxy for cigarette smoking [31]. In addition, the occurrence of cirrhosis might impact the prognosis of HCV patients and IBT is used with caution in patients with cirrhosis and is contraindicated in patients with decompensated liver disease [4]. We clarified the etiology and severity of liver disease by code as follows: cirrhosis of the liver without mention of alcohol (ICD-9-CM: 571.5, 571.6), alcoholic liver cirrhosis (ALC) (571.2), other alcoholic liver diseases (571.0, 571.1,571.3), and liver cirrhosis-related complications including ascites (789.5), esophageal varices (456.0, 456.1, 456.2) and hepatic encephalopathy (572.2). Covariates included all variables shown in Table 1, in which diseases were coded as yes/no and defined by ICD-9-CM codes.

**Table 1:** **Characteristics of the enrolled patients**

Variable	Interferon-based therapy		*P* value
	Yes	No	
	N = 621	N = 2460	
**Sex, n (%)**			0.849
Male	351 (56.5)	1380 (56.1)	
Female	270 (43.5)	1080 (43.9)	
**Age, n (%)**			0.999
20 – 29	22 (3.5)	84 (3.4)	
30 – 39	54 (8.7)	217 (8.8)	
40 – 49	123 (19.8)	485 (19.7)	
50 – 59	231 (37.2)	906 (36.8)	
60 – 69	154 (24.8)	620 (25.2)	
≧70	37 (6.0)	148 (6.0)	
**Comorbid disease, n (%)**			
Human immunodeficiency virus infection	2 (0.3)	12 (0.5)	0.583
Chronic obstructive pulmonary disease	41 (6.6)	128 (5.2)	0.171
Connective tissue disease	182 (29.3)	702 (28.5)	0.704
Silicosis	1 (0.2)	1 (0.04)	0.615
Diabetes mellitus	126 (20.3)	449 (18.3)	0.244
End stage renal disease	7 (1.1)	83 (3.4)	*0.003
Malignancy	38 (6.1)	102 (4.1)	*0.035
**Liver disease, n (%)**			
Non-alcoholic liver cirrhosis	99 (15.9)	214 (8.7)	*<0.001
Alcoholic liver cirrhosis	7 (1.1)	27 (1.1)	0.949
Other alcoholic liver disease	11 (1.8)	28 (1.1)	0.207
**Liver disease severity, n (%)**			
Ascites	4 (0.6)	19 (0.8)	0.740
Esophageal varices	4 (0.6)	20 (0.8)	0.668
Hepatic encephalopathy	0 (0)	16 (0.7)	*0.043

*means *P* < 0.05.

### Statistical analysis

All the data processing and statistical analyses were performed with SAS 9.3 software (Cary, NC, USA). Chi-square tests were used to compare the distributions of categorical variables between patients who did or did not receive IBT. The time-to-event analysis involved estimating the probability that an event would occur at different points in time. The endpoint of follow-up in the subjects developing active TB was the date of 1) having taken two anti-TB medications for more than 90 days, and 2) having a TB-specific ICD-9 code, and those lost to follow-up were censored on the date of last visit, creating “censored” data. The most common time-to-event statistical methods are Kaplan-Meier analysis and the proportional hazards model. The Kaplan-Meier analysis was computed to estimate the difference in the hazard ratio of TB development between both cohorts. The proportional hazards model was applied to analyze the effect of single and multiple covariates in predicting whether TB developed. Both short-term (1 year after index date) and long-term (9 years after index date) follow-up were included for analysis in this study.

## Results

### Baseline characteristics of the study population

Among the 699 HCV patients who received IBT, 621 patients (88.84%) were treated for a minimum of 2 months and were therefore eligible as the IBT-treated cohort (n = 621). The control cohort comprised 2,460 untreated patients who were selected from those not receiving IBT (Figure 1). Table 1 shows the baseline characteristics of the two cohorts. Most patients in both cohorts were aged between 50 to 69 years. To compare with the untreated cohort, patients in IBT-treated cohort had more malignancy and non-alcoholic liver cirrhosis. (*P* = 0.035 and <0.001, respectively) Patients in the untreated cohort had more ESRD and hepatic encephalopathy (*P* = 0.003 and 0.043, respectively).

### Incidences of TB among the two cohorts

**Figure 2:**
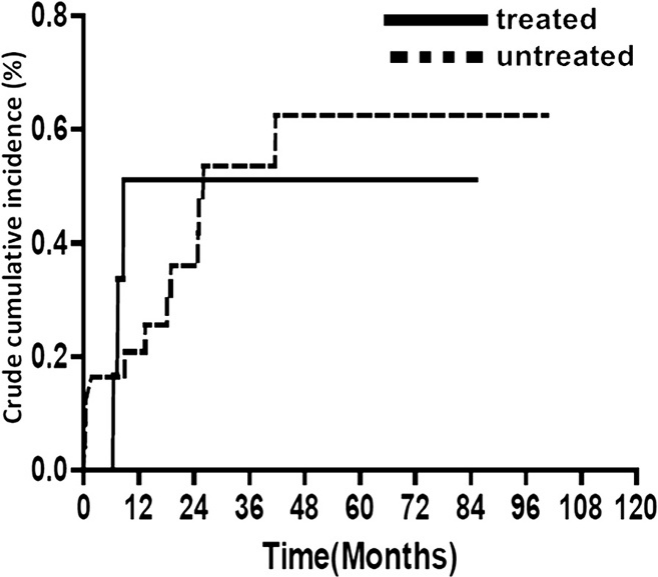
**1 minus Kaplan Meier to approximate cumulative incidence of tuberculosis among the IBT cohort (treated) and the untreated cohort (untreated) during 1-year follow-up (log rank test: 1.26,** *P*  **= 0.261).**

During the 9-year enrollment period, the treated and untreated cohorts were followed up for a mean (± SD) duration of 6.97 ± 0.02 years and 8.21 ± 0.01 years, respectively. Among the treated cohort, which included those who had ever received IBT for a minimum of 8 weeks, the mean (± SD) duration of the antiviral regimen was 20.29 ± 4.50 weeks. During a 1-year follow-up, 3 patients developed TB in IBT treated cohort and 5 patients developed TB in untreated cohort. During the long-term follow-up, 3 patients developed TB in IBT- treated cohort and 12 patients developed TB in untreated cohort. The cumulative incidences of TB during this study period were 0.150 and 0.151 per 100 person-years in the IBT treated and untreated cohorts in long-term follow-up, respectively. There was no significant difference in the incidence of active TB in either cohort during a 1-year follow-up (Adjusted Hazard Ratio (AHR): 2.81, 95% Confidence Interval (95% CI): 0.61–12.98) or the long-term follow-up (AHR: 1.02, 95% CI: 0.28 – 3.78) (Table 2). Further analysis by the log-rank test revealed no significant difference of the incidence of TB in both cohorts during 1-year and long-term follow-up periods.(*P* = 0.261 and 0.987, respectively) Figure 2 showed the crude cumulative incidence of tuberculosis among both cohorts in 1-year follow-up.

**Table 2:** **Results of hazard ratios in IBT-treated cohorts in 1-year and long-term follow-up**

Cohort	TB case n. (%)	Crude HR (95% CI)	Adjusted HR (95% CI)
	1-year follow-up		
Control (n = 621)	3 (0.5)	1	1
IBT-treated (n = 2640)	5 (0.2)	2.36(0.57-9.89)	2.81(0.61-12.98)
	Long-term follow-up		
Control (n = 621)	3 (0.5)	1	1
IBT-treated (n = 2640)	12 (0.5)	0.99(0.28-3.50)	1.02(0.28-3.78)

### Multivariate-adjusted association of antiviral therapy with active TB

The Cox proportional hazards model demonstrated that IBT was not associated with active TB (Tables 3 and 4). The only factor associated with the development of active TB was the occurrence of hepatic encephalopathy. (Hazard Ratio (HR):54.90 and 95% CI: 2.82–1069.53 in 1-year follow-up; HR: 16.75 and 95% CI: 1.22 – 231.02 in long-term follow-up).

**Table 3:** **Results of the multivariate-adjusted cox proportional hazards model for active tuberculosis cases in 1-year follow-up**

	Adjusted hazard ratio	95% confidence interval	*P* value
Male	2.75	0.55 –13.79	0.218
Age	1.07	0.99 – 1.15	0.103
Comorbidity			
Human immunodeficiency virus infection	-	-	-
Chronic obstructive pulmonary disease	-	-	-
Connective tissue disease	0.33	0.04- 2.78	0.309
Silicosis	-	-	-
Diabetes mellitus	2.81	0.62 – 12.75	0.180
End stage renal disease	-	-	-
Malignancy	1.78	0.20 – 15.69	0.602
Liver disease			
Non-alcoholic liver cirrhosis	0.97	0.11–8.46	0.976
Alcoholic liver cirrhosis	-	-	-
Other alcoholic liver disease	-	-	-
Liver disease severity			
Ascites	-	-	-
Esophageal varices	-	-	-
Hepatic encephalopathy	54.90	2.82–1069.53	*0.008
Interferon-based therapy	2.81	0.61–12.98	0.185

*means *P* < 0.05.

**Table 4:** **Results of the multivariate-adjusted cox proportional hazards model for active tuberculosis cases in long-term follow-up**

	Adjusted hazard ratio	95% confidence interval	*P* value
Male	2.41	0.75 – 7.72	0.139
Age	1.05	0.99 – 1.1	0.102
Comorbidity			
Human immunodeficiency virus infection	-	-	-
Chronic obstructive pulmonary disease	0.77	0.09 – 6.36	0.809
Connective tissue disease	0.36	0.08- 1.65	0.189
Silicosis	-	-	-
Diabetes mellitus	2.01	0.67 – 6.06	0.216
End stage renal disease	1.97	0.25 – 15.63	0.522
Malignancy	0.99	0.11 – 8.58	0.993
Liver disease			
Non-alcoholic liver cirrhosis	1.48	0.30 – 7.40	0.631
Alcoholic liver cirrhosis	-	-	-
Other alcoholic liver disease	-	-	-
Liver disease severity			
Ascites	-	-	-
Esophageal varices	-	-	-
Hepatic encephalopathy	16.75	1.22 – 231.02	*0.035
Interferon-based therapy	1.02	0.28 – 3.78	0.974

*means *P* < 0.05.

## Discussion

In this population-based cohort study, IBT was not associated with active TB in HCV patients after adjustment for possible confounding factors, such as HIV infection, silicosis, COPD, connective tissue disease and malignancy. During the 1-year and long-term follow-up periods, the crude cumulative TB incidences in both cohorts were not significantly different. The incidence of TB in the IBT treated cohort was 0.150 per 100 person-years during this study period, which was lower than the reported incidence rates of HIV-HCV co-infected patients receiving IBT (0.7 cases per 100 person-years) in Spain and HCV patients (1.4 cases per 100 person-years) in Pakistan [32],[33].

The role of IFNs in exacerbating TB infection remains controversial. Few case reports have described the association between IBT and active TB [18]-[22]. TB events in clinical trials of HCV patients treated with IBT were rarely reported. Only one study in Taiwan [34] reported 308 treatment-naive HCV-1–infected patients receiving IBT resulted in one case with TB reactivation at week 32 of IBT.

Three HCV infected patients with active TB were identified in the IBT-treated cohort in this study. These TB cases were confirmed within 38 weeks of IBT initiation. IBT was not an independent risk factor for the development of active TB in our study; the only risk factor reported was advanced liver disease. Because of the multiple levels of immune dysfunction, cirrhotic patients are predisposed to infectious diseases, including bacterial and TB infection [35]. In Taiwan, liver cirrhosis and chronic liver disease were significant risk factors associated with death in a TB infected population [36]. Our results demonstrate that the association of hepatic encephalopathy and TB may indicate a vulnerability to TB for cases with compromised liver function. Further prospective study is necessary to clarify the role of IBT in active TB cases during the hepatitis treatment course.

The HCV shares risk factors and routes of transmission with some infectious diseases. Compared to people without HCV infection, HCV carriers were significantly associated with many infectious diseases, including TB [37]. It is presumed that HCV itself is a risk factor for TB infection regardless of whether the HCV was treated with IBT. Although IBT is not associated with increasing hazard of active TB in HCV infected patients in our study, it seemed to be higher among IBT group with one-year follow-up. It is reasonable to be careful the occurrence of active TB while using IBT to treat HCV infected patients in one-year period. Further study to evaluate this issue by enrolling greater sample size is necessary.

Several limitations of this study warrant discussion. First, although we did not demonstrate an association between IBT and active TB in this study, this lack of association maybe related to the different TB prevalences in the different age populations in our country. In the IBT treated cohort, 69.2% (430/621) of patients were less than 60 years old. However, the TB incidence rate generally increases in older population, especially in those over 65 years old [24]. IBT in HCV infected patients in the elderly population needs further investigation. Second, the Taiwan NHIRD did not contain direct laboratory results. Therefore, we were unable to determine how viral genotype, viral load and CD4 numbers might influence the outcomes. Because of the limitation of this database, there was no standard procedure for diagnosis of TB infection (acid-fast stain, culturing and histopathology). Instead, we defined TB infection according to ICD-9-CM codes of TB with taking anti-TB medication for more than 3 months. It is likely that some patients with TB infections did not submit claims for medication, or died before 3 months of treatment, which may underestimate the total number of MTB infected patients. Third, some patients were treated with corticosteroids, which are known to increase the risk of TB [38]. Although we adjusted for patients with connective tissue diseases and COPD, a proportion of the patients exposed to corticosteroids would be underestimated. Finally, this study lacked information on several important risk factors, such as smoking, nutritional status, intravenous drug abuse, and occupational exposure, which are also not available in the NHIRD.

## Conclusion

Our results showed that IBT is associated with increased hazard of active TB in HCV infected patients in 1-year follow-up; however, the effect sizes were not statistically significant.

## Authors’ original submitted files for images

Below are the links to the authors’ original submitted files for images. Authors’ original file for figure 1 Authors’ original file for figure 2

## Abbreviations

TB: tuberculosis
IFN: Interferon
IBT: interferon-based therapy, COPD, chronic obstructive pulmonary disease
HCV: Hepatitis C virus
IBT: interferon-based therapy
IFNs: Interferons
LHID: longitudinal Health Insurance Database
NHI: National Health Insurance
TB: tuberculosis
