# Supplementary figures and images for: Incidence rates of tuberculosis in chronic hepatitis C infected patients with or without interferon based therapy: a population-based cohort study in Taiwan
Source: BMC Infect Dis. 2014 Dec 19;14:705. doi: 10.1186/s12879-014-0705-y (PMC4307221; doi:10.1186/s12879-014-0705-y)

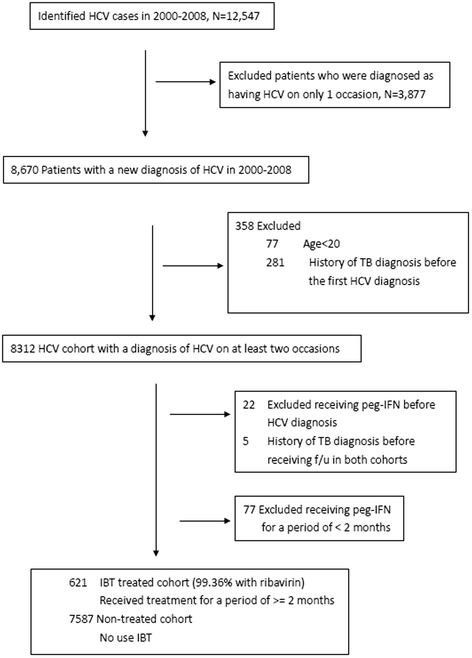

Supplement: Supplementary file 1 — Authors’ original file for figure 1 [file 12879_2014_705_MOESM1_ESM.gif]

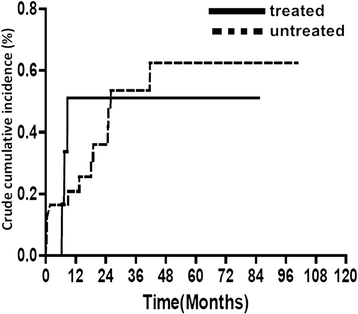

Supplement: Supplementary file 2 — Authors’ original file for figure 2 [file 12879_2014_705_MOESM2_ESM.gif]
